# Prebiotic Bimuno^®^ GOS reduces illness symptoms and supports gut barrier function and immunity after intermittent exercise in the heat

**DOI:** 10.1113/EP092682

**Published:** 2026-03-24

**Authors:** Connor J. Parker, Samantha J. Abbott, Luke R. Butterfield, Kirsty A. Hunter, Michael A. Johnson, Graham R. Sharpe, Neil C. Williams

**Affiliations:** ^1^ Department of Sport Science, School of Science and Technology Nottingham Trent University Nottingham UK

**Keywords:** gastrointestinal discomfort, intestinal epithelial injury, prebiotic, salivary IgA, upper respiratory symptoms

## Abstract

Exercise in hot climates increases the risk of gastrointestinal (GI) disruption and respiratory illness. We investigated the effects of a 42 day prebiotic intervention on markers of intestinal epithelial injury, GI discomfort and immunity following football specific exercise in the heat and daily upper respiratory illness. Twenty‐six team‐sport male athletes were randomized to receive 3.65 (2.75 active galactooligosaccharide) g day^−1^ of either Bimuno galactooligosaccharide (Bimuno^®^ GOS; *n* = 13) or a maltodextrin placebo (Placebo; *n* = 13) for 42 days in a double‐blind parallel group design. At days 0 and 42 of each intervention, participants completed the football specific intermittent treadmill protocol in 33°C and 50% relative humidity. Blood, saliva and GI symptoms were collected at days 0 and 42 for the assessment of plasma intestinal fatty acid binding protein, lipopolysaccharide binding protein, salivary immunoglobulin A (sIgA) and GI discomfort. Participants also completed questionnaires for self‐reported upper respiratory and gastrointestinal symptoms (daily and weekly, respectively), over the 42 days. At day 42, there was a reduction in pre‐full‐time change in plasma intestinal fatty acid binding protein and severity of GI discomfort in the Bimuno^®^ GOS group compared with Placebo, but no change in lipopolysaccharide binding protein. The day 0–42 change in sIgA secretion rate after the football specific intermittent treadmill protocol was higher in the Bimuno^®^ GOS than the Placebo group, and during the 42 days there was a reduction in upper respiratory symptom duration and severity in the Bimuno^®^ GOS group compared with Placebo. In team‐sport athletes, 42 days of supplementation with prebiotic Bimuno^®^ GOS can alleviate GI disruption and better maintain sIgA secretion rate in response to football specific activity in the heat, whilst also reducing the duration and severity of upper respiratory symptoms.

## Abstract (highlights)

- **What is the central question of the study?**
  Does a 42 day prebiotic intervention reduce gastrointestinal injury and symptoms and support respiratory health during intermittent exercise in the heat?
- **What is the main finding and its importance?**
  There was a reduction in pre‐full‐time change in intestinal fatty acid binding protein and gastrointestinal symptoms during a simulated football match in the heat after the 42 day prebiotic intervention but not placebo, indicating less intestinal epithelial injury. This was accompanied by a reduction in duration and severity of upper respiratory symptoms in the prebiotic group. Hence, a 42 day prebiotic intervention can support gastrointestinal function and immune and respiratory health of individuals undergoing intermittent exercise in the heat.

## 1 INTRODUCTION

Exercise in the heat can acutely compromise gastrointestinal (GI) and immune function, as shown by the onset of GI discomfort and decreases in the secretion rate of the main antibody in saliva, secretory immunoglobulin A (sIgA) (Laing et al., 2005; Yeh et al., 2013). Although, the exact mechanisms of exercise‐induced gastrointestinal symptoms (GIS) are not clear, it is likely that splanchnic hypoperfusion and intestinal ischaemia contribute, leading to intestinal epithelial cell injury and hyperpermeability (van Wijck et al., 2011); dependent upon the resultant gut wall integrity, this allows for translocation of pathogenic organisms from the lumen into circulation, leading to a systemic inflammatory response.

Reductions in sIgA are commonly associated with an increased susceptibility to upper respiratory symptoms (URS) in athletes (Gleeson et al., 1999; Nieman & Nehlsen‐Cannarella, 1991). Prolonged exercise in cool and hot environments has been shown to reduce sIgA secretion rate (Laing et al., 2005; Walsh et al., 2002), with an increase in sympathetic nervous system activity and vasoconstriction of the blood supply to the salivary glands proposed as the mechanistic cause (Chicharro et al., 1998). Exercising in the heat is reported to induce a greater noradrenaline response and higher sympathetic activity than when performed in thermoneutral conditions (Galbo et al., 1979), possibly leading to a greater reduction in sIgA secretion rate and a higher risk of URS.

It is evident that an acute bout of continuous or intermittent exercise can result in acute immunosuppression and increases in intestinal epithelial injury and symptoms (Gaskell et al., 2022, 2021; McKenna et al., 2022; Pugh et al., 2017, 2019; Yeh et al., 2013). Recent literature suggests that intermittent, invasion team‐based sports can also compromise GI and immune function, as demonstrated by elevated levels of plasma intestinal fatty acid binding protein (I‐FABP) in elite rugby union and football players (Chantler et al., 2022; Clayton et al., 2024) and reporting of GIS and URS throughout the course of a competitive elite season (Parker et al., 2023). Concerningly, it is increasingly common that team‐based sports are performed in hotter climates. Exercising in hotter environments provides further stress to both the GI and respiratory tracts compared with thermoneutral environments, leading to greater epithelial injury and a higher risk of upper respiratory illness (Snipe et al., 2018; Yeh et al., 2013). Therefore, investigations into dietary biotic solutions that can reduce the likelihood of illness and discomfort during such performances are warranted.

The human gut microbiota harbours trillions of bacteria that contribute significantly to GI integrity and function (Eckburg et al., 2005). Common dietary interventions to target the gut microbiome include probiotics, prebiotics, synbiotics and postbiotics. A recent systematic review found that probiotic and synbiotic supplementation increases the relative abundance of supplemented microbial species and strains but does not significantly affect GI integrity or short‐chain fatty acid concentrations in healthy, active adults (Rauch et al., 2022). Alternatively, prebiotics, defined as substrates that are selectively used by host microorganisms conferring a health benefit (Gibson et al., 2017), might influence GI integrity at rest (Russo et al., 2012). One benefit of using prebiotics rather than probiotics is their ability to act at the genus level, which bypasses the species variability of probiotics. It has recently been demonstrated that a 24 week intervention with a low‐dose prebiotic, Bimuno galactooligosaccharide (Bimuno^®^ GOS; 2.75 g day^−1^) reduced the duration of URS and the severity and incidence of GIS in elite rugby union players during a competitive playing season, improving player availability for training and competition, possibly owing to improvement in sIgA secretion rate (Parker et al., 2023). Although these findings are novel and might have a significant impact on athlete availability, the design of the study did not allow for the assessment of GI functioning, immunity and symptoms in response to a specific exercise stressor. Recently, Rauch et al. (2025) have demonstrated, for the first time, that a 16 g day^−1^ prebiotic mixture for 8 weeks moderately attenuated the increase in intestinal epithelial injury during prolonged exercise in the heat. However, the effects of a lower dose of prebiotic GOS on gut barrier function and immunity during intermittent exercise in the heat are unknown.

Therefore, the aim of the present study was to assess the effects of a 6 week prebiotic Bimuno^®^ GOS supplementation (2.75 g day^−1^) on markers of exercise‐induced intestinal epithelial injury, GIS, sIgA and URS following football specific exercise in the heat. It was hypothesized that Bimuno^®^ GOS supplementation would reduce post‐exercise intestinal epithelial injury and GI symptoms whilst also alleviating the decrement of sIgA. Furthermore, it was hypothesized that 6 weeks of Bimuno^®^ GOS would reduce the incidence, severity and duration of URS.

## 2 MATERIALS AND METHODS

### 2.1 Ethical approval

The study was approved by the Nottingham Trent University Human Invasive Ethics Committee (application number 702), and all experimental procedures were performed in accordance with the *Declaration of Helsinki*. Each participant signed a written informed consent document before beginning the study.

### 2.2 Participants and study design

**FIGURE 1:**
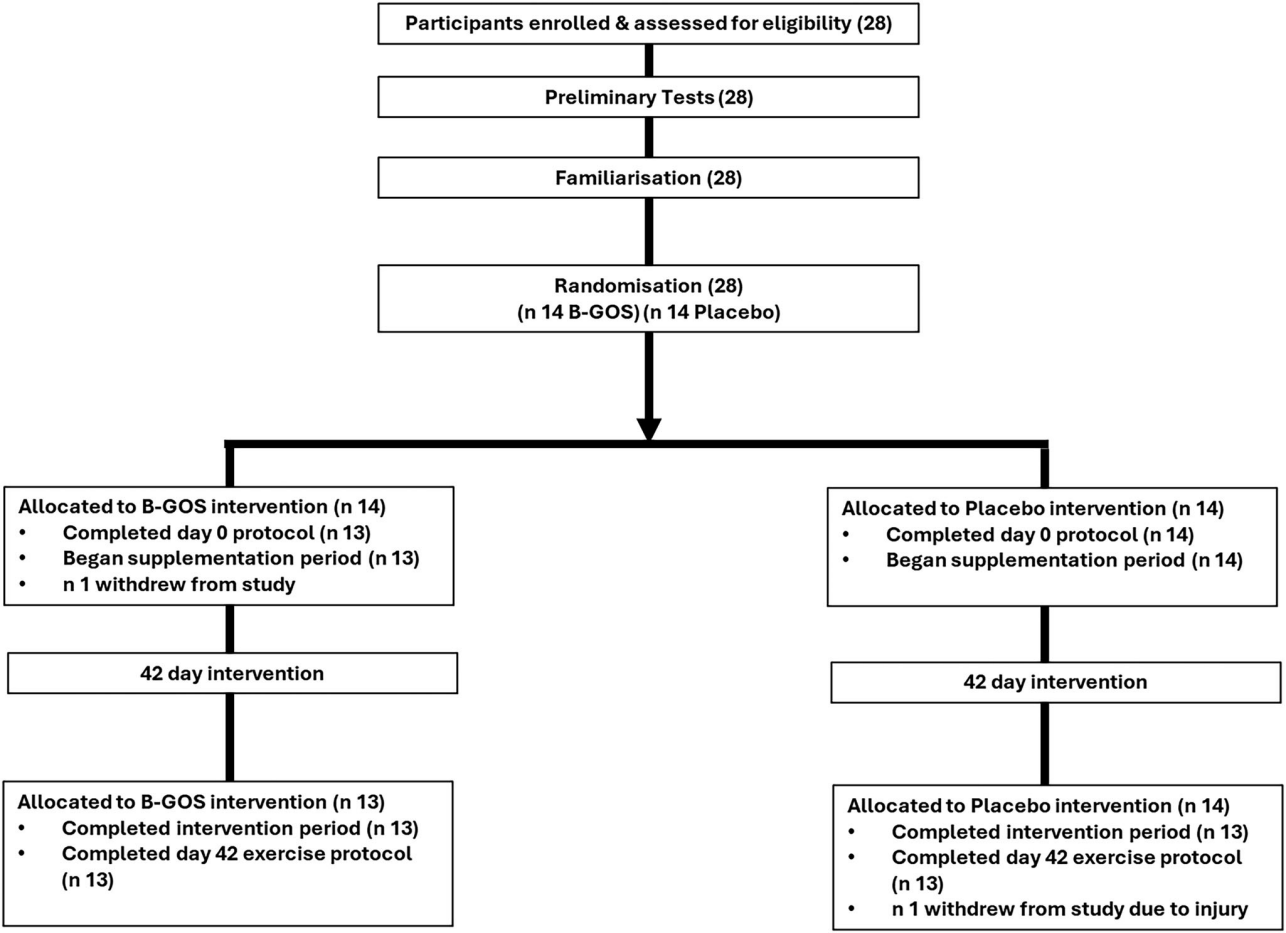
Overview of study design. Abbreviation: B‐GOS, Bimuno‐galactooligosaccharide.

A total of 26 male recreationally active team‐sport players [age 23.6 ± 3.2 years, height 182.2 ± 6.9 cm, body mass 79.83 ± 9.28 kg and maximal oxygen uptake (${\dot{V}}_{O_{2}\max}$) 53.6 ± 4.7 mL kg^−1^ min^−1^] participated in the present study using a double‐blind, randomized, placebo‐control design (Figure 1). All participants were non‐smokers, had no history of GI illness (e.g., irritable bowel syndrome, inflammatory bowel disease, lactose intolerance and chronic constipation or diarrhoea) and were not regularly taking medication or consuming supplements or foods enriched with probiotics, prebiotics, synbiotics, postbiotics or vitamins. To be able to participate, participants were required to perform ≥4 h of team‐sport activity (e.g., football, rugby or hockey) per week.

### 2.3 Overview of experimental design

All data were collected at Nottingham Trent University, with each participant visiting the laboratory on four separate occasions between October and April, thereby excluding the UK summer months to minimize potential heat acclimatization effects. Following a baseline visit, each participant completed a familiarization visit prior to two experimental trials, which were performed at day 0 (D0) and day 42 (D42) of the dietary intervention. The familiarization and two experimental trials were performed in an environmental chamber (TISS series 201003‐1, TIS services UK) set at 33°C and 50% relative humidity. Following initial recruitment, participants were adaptively randomized based on age and body mass to ensure homogeneity between groups and randomized to consume either the Bimuno^®^ GOS prebiotic or placebo supplement daily. The D0 and D42 visits were completed at the same time of day, and participants were instructed to arrive 2 h postprandial and having consumed 500 mL of water 2 h prior to arrival. In the 24 h preceding each experimental trial, participants consumed the same diet and were instructed to avoid exercise, alcohol, non‐steroidal anti‐inflammatory drugs and caffeine intake. Participants ingested the final dose of supplement 24 h before the visit. Throughout the intervention period, participants were instructed to avoid significant changes in training and dietary patterns and the introduction of new dietary supplements.

### 2.4 Baseline testing and familiarization

During the first visit, participants performed a speed lactate test and an incremental exercise test to failure to obtain their maximal lactate steady state and ${\dot{V}}_{O_{2}\max}$. Following a 5 min rest, a baseline capillary sample was collected from the fingertip for the analysis of blood lactate (Biosen lactate analyser, EKF Diagnostics, Germany). Body mass, height and resting heart rate (HR) (Beurer PM62, Beurer, Germany) were then measured. To start the speed lactate test, participants began running on a motorized treadmill (HP Cosmos, Germany) at a speed of 9 km h^−1^ at a 1% gradient for 3 min. At the end of each 3 min stage, the participants straddled the treadmill, and a capillary blood sample was collected (MacLeod et al., 2018). The speed then increased by 1 km h^−1^ until the maximal lactate steady state threshold was achieved, which was defined as the fastest speed with a <1 mmol L^−1^ increase in blood lactate above the preceding levels (Astrand et al., 2003).

After a 10 min rest, participants started the ${\dot{V}}_{O_{2}\max}$ test. Running was initiated at their maximal lactate steady state at a 1% gradient, with an increase of 1 km h^−1^ every minute until task failure. Ventilatory and pulmonary variables were measured breath‐by‐breath throughout the maximal test using a metabolic cart (Vyntus CPX, Vyaire Medical Inc., UK), which was calibrated with the known gases (O_2_ = 15.96%, CO_2_ = 4.99%). Participants wore a facemask for the entirety of the test (Hans Rudolph 7450, Hans Rudolph Inc., USA), and expired air was sampled using a digital volume transducer, and O_2_ and CO_2_ sensors. Ten‐second averages for the ventilatory and pulmonary variables were calculated, with ${\dot{V}}_{O_{2}\max}$ considered the highest value of oxygen uptake over any 10 s average.

Together, the results from the speed lactate and ${\dot{V}}_{O_{2}\max}$ test provided the speeds used for the stages of the football specific intermittent treadmill protocol (FSITP). At least 1 week after the baseline visit, participants completed the familiarization trial and performed one half of the FSITP in 33°C, 50% relative humidity.

### 2.5 Experimental visits

**FIGURE 2:**
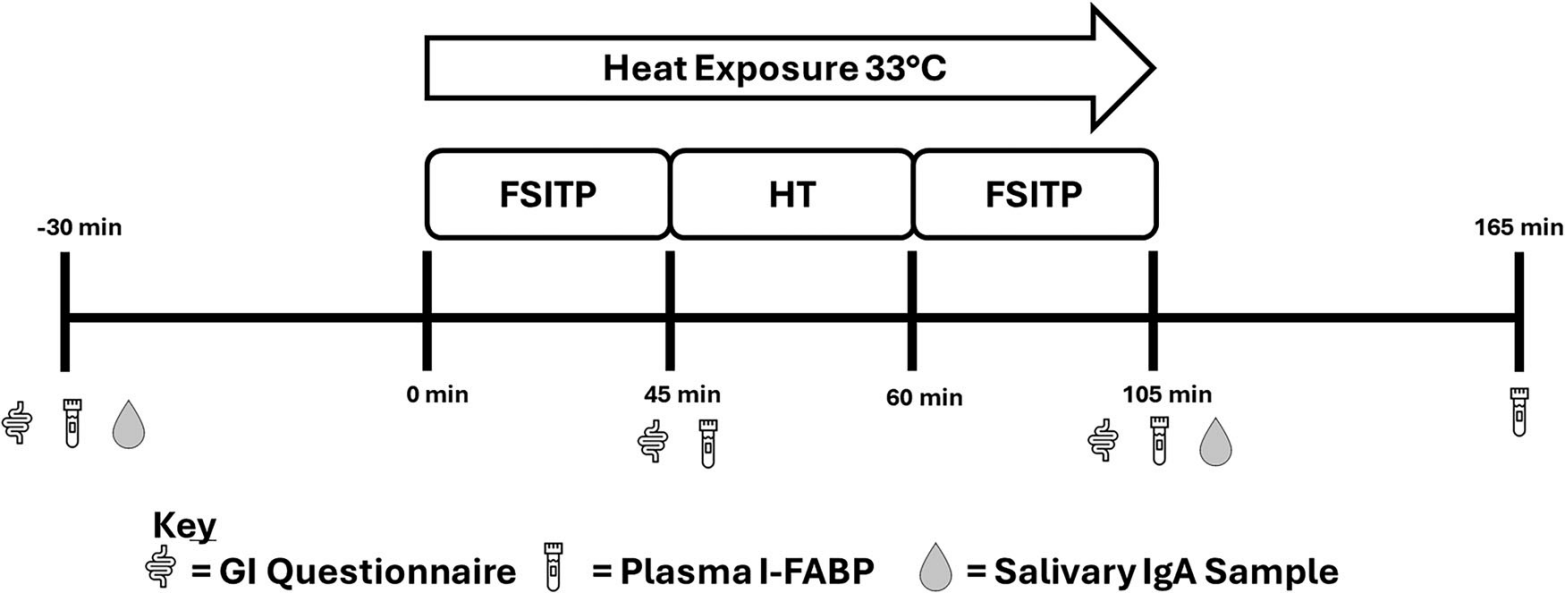
Schematic diagram of main experimental trial on days 0 and 42. Abbreviations: FSITP, football specific intermittent treadmill protocol; GI, gastrointestinal; HT, half‐time; I‐FABP, intestinal fatty acid binding protein; IgA, immunoglobulin A.

Participants arrived at the laboratory and provided a urine sample for the assessment of hydration status via urine osmolality (Pocket‐Pal Osmo‐Osmocheckä, 4595‐E04, Vitech Scientfic Ltd, Horsham, UK). A value of <800 mosmol kg^−1^ was considered hydrated (Perrier et al., 2015). If a sample was >800 mosmol kg^−1^, the participant would be instructed to consume water and rest for 30 min, and another sample was collected. Following this, nude body mass (GFK 150 AEADAM digital scale, Vitech Scientific Ltd) was measured in private. Participants then rested for 10 min before samples of venous blood, capillary blood and saliva were collected. Resting HR and core temperature (*T*_core_) were then collected. The *T*_core_ was determined via an ingestible telemetric pill (e‐celsius BodyCap, France), which was ingested 6 h prior to the trial. Participants then completed the 90 min FSITP in the heat chamber (33°C and 50% relative humidity). The FSITP was made up of two 45 min blocks of activity, separated by a 15 min rest period to replicate half‐time (Figure 2). A 3 min water break was provided halfway through each half, when participants could drink water ad libitum. The amount of water consumed was recorded and repeated for the subsequent trial. Throughout the FSITP, HR, *T*_core_ and the rating of perceived exertion (RPE; Borg et al., 1982) were recorded every 5 min. Venous and capillary blood samples were collected at half‐time. Thermal sensation and fatigue were assessed using subjective questionnaires every 15 min (Micklewright et al., 2017; Young et al., 1987). Immediately after completion of the FSITP, nude body mass, urine, venous, capillary and saliva samples were collected. A final venous blood sample was collected after 60 min of rest. Each participant completed a gastrointestinal symptom questionnaire (Gaskell et al., 2019) before, at half‐time and on completion of the FSITP. Following completion of the D0 experimental trial, the participants began the 42 day intervention of either Bimuno^®^ GOS or placebo. At D42, participants returned to repeat the experimental trial.

### 2.6 FSITP

The entire FSITP protocol was performed on a motorized treadmill at a gradient of 1%. The treadmill speeds were customized and determined by the participant's speed lactate and ${\dot{V}}_{O_{2}\max}$ results. A total of seven different speeds were incorporated into the protocol to simulate the intensity, accelerations and decelerations of a football match. The speeds included stationary (0 km h^−1^) walking (4 km h^−1^), jogging (speed before maximal lactate steady state), low speed (85% ${\dot{V}}_{O_{2}\max}$), moderate speed (100% ${\dot{V}}_{O_{2}\max}$), fast run (21 km h^−1^) and sprint (25 km h^−1^).

### 2.7 Supplementation

Participants were match‐randomized to consume either 3.65 g day^−1^ (containing 2.75 g active GOS) of dietary prebiotic Bimuno galactooligosaccharide (Bimuno^®^ GOS; Clasado Ltd, Reading, UK) or 3.65 g day^−1^ of placebo (Placebo; maltodextrin). Both supplements were provided as single‐dose sachets in powdered form and were identical in taste and colour. Supplements were blinded at the site of manufacture (provided by Clasado Ltd) and were not revealed to researchers until all data had been collected and statistically analysed. Participants were instructed to consume the supplement mixed in water at breakfast. Each participant returned used and unused sachets to confirm supplement compliance throughout the 42 day period.

### 2.8 Collection and analysis of blood biomarkers

At D0 and D42, all participants provided blood samples prior (Pre), at half‐time, full‐time and 60 min post (Post60) the FSITP for the determination of plasma concentrations of I‐FABP and lipopolysaccharide binding protein (LBP). Samples were obtained from the antecubital vein in three 6 mL vacutainers, two containing an EDTA anticoagulant and one containing a heparin anticoagulant (BD Vacutainer^®^). Samples were centrifuged immediately, with plasma aliquoted into clean Eppendorf tubes and frozen at −80°C until further analysis. I‐FABP and LBP were assessed using commercially available ELISA (Hycult Biotech, Amsterdam, The Netherlands). For I‐FABP, the intra‐ and interassay variation was 2.7% and 3.4%, respectively. For LBP, the intra‐ and interassay variation was 3.1% and 4.8%, respectively.

### 2.9 Collection and analysis of saliva

At D0 and D42, saliva samples were collected before and immediately after the FSITP to determine sIgA. Participants were instructed to rinse their mouth with plain water, then rest for 10 min before producing a saliva sample via the passive drool method for 2 min. Samples were weighed and immediately frozen at −80°C until further analysis. The sIgA concentrations were determined by a commercially available ELISA (Salimetrics, Philadelphia, PA, USA). The intra‐ and interassay variation was 1.7% and 2.6%, respectively. Owing to the influence of flow rate on sIgA concentrations, sIgA secretion rate was calculated. The sIgA concentration (in micrograms per millilitre) was multiplied by the flow rate (volume/duration), resulting in a concentration measure per unit of time (in micrograms per minute).

### 2.10 GIS during FSITP

To assess the presence and severity of GIS during the FSITP, participants completed a GIS questionnaire before commencing the protocol, at half‐time and again at full‐time (Gaskell et al., 2019). Participants were educated on the questionnaire and were advised to rate the presence of each symptom over each 45 min period using a 10‐point visual analog scale, with 1–4 indicative of mild GIS (not substantial enough to interfere with exercise), 5–9 indicative of severe GIS (substantial enough to interfere with exercise), and 10 indicating extremely severe GIS (warrants exercise reduction or cessation). If no specific GIS was reported, this was rated as zero. The questionnaire was adapted to incorporate 14 GIS, categorized as those associated with upper GIS, lower GIS and other GIS. The rating for each symptom was summed to give a total for each 45 min period. The scores for the two 45 min periods were then summed to provide an overall score during the FSITP. This process was repeated for the symptoms exclusive to the upper GI tract and lower GI tract and to those that are associated with neither and are simply categorized as ‘other symptoms’ (Gaskell et al., 2019).

### 2.11 URS and GIS during supplementation

To assess the incidence and severity of URS during the supplementation period, participants completed the Jackson questionnaire daily (Jackson et al., 1958). Participants would state whether an upper respiratory illness was present and rated the severity of eight symptoms (headache, chilliness, sneezing, sore throat, malaise, cough, nasal discharge and nasal obstruction) over each 24 h period. Each symptom was rated on a scale of 0–3 (0 = none; 1 = mild; 2 = moderate; and 3 = severe). Symptom scores for each day were summed to give a total Jackson score. A URS episode was defined using the Jackson criteria as applied by Martineau et al. (2015), i.e., any period lasting ≥3 days with a Jackson score of ≥14 and the presence of nasal discharge, or a symptom score of <14 with a subjective impression of having a cold for ≥3 days. If URS returned within 1 week, it was regarded as the same episode. Participants were asked to report the use of any over‐the‐counter cold and flu medication, but would have been excluded if they reported being prescribed medication during the intervention period.

To determine the incidence and severity of GIS during the supplementation period, participants completed the gastrointestinal symptom rating scale (GSRS) weekly. Participants were asked to rate the presence and severity of 15 GIS during the previous 7 days on a seven‐point Likert scale (1 = absent; 2 = minor; 3 = mild; 4 = moderate; 5 = moderately severe; 6 = severe; and 7 = very severe) (Svedlund et al., 1988). The symptoms included upper abdominal pain, epigastric pain, heartburn, regurgitation, abdominal rumbling, bloating, nausea, empty feeling in the stomach, early satiety, postprandial fullness, belching, flatulence, haematemesis, dysphagia and questions on defecation. In analysis, a rating of ≥2 defined symptom presence and ≥4 defined moderate discomfort from that symptom. Baseline values were from the 7 days preceding D0.

### 2.12 Statistical analysis

After normal distribution was confirmed, a mixed‐model ANOVA [with group as a between‐subjects factor and with day (two levels) and time (four levels) as repeated/within‐subjects factors, with <0.01, <0.06 and >0.14 considered as a small, medium, and large effect] was used to assess any changes in absolute I‐FABP, LBP and sIgA. To gain a better scope of the changes following the intervention, the pre‐full‐time exercise change (Δ) was calculated for plasma I‐FABP at both D0 and D42, by subtracting the pre‐exercise value from the value observed at full‐time, as previously described (McKenna et al., 2022; McKenna et al., 2022). Given that data were not normally distributed, a Kruskal–Wallis test was used to assess any between‐group differences in pre‐full‐time Δ plasma I‐FABP. To assess any changes in sIgA secretion rate, the pre‐exercise value was subtracted from the full‐time value to gain a pre–post FSTIP change for both D0 and D42. The pre–post value at D0 was then subtracted from the D42 pre–post value to show a relative change following the Bimuno^®^ GOS and placebo interventions. To assess the impact of the intervention on weekly GIS, the D0 value was subtracted from D42. Student's unpaired *t*‐test was used to assess any between‐group difference for both sIgA and GIS. All physiological data (HR, *T*_core_, blood lactate, RPE, thermal sensation, fatigue, urine osmolality, body mass and sweat rate) were checked for normal distribution, then analysed using a mixed‐model ANOVA (with intervention as a between‐subjects factor and time as a repeated/within‐subjects factor, with <0.01, <0.06 and >0.14 considered as a small, medium and large effect, respectively]. GI symptoms during exercise were categorized into four categories (total, upper, lower and other). The symptom scores that were collected at half‐time and full‐time were summed to provide an overall score for each category. The percentage change from D0 to D42 was calculated for each category, and between‐group differences were assessed using a Mann–Whitney *U*‐test. All URS data were analysed with a Mann–Whitney *U*‐test. The statistical significance was accepted at the 95% confidence level (*P* < 0.05). The mean and SD were used to describe the average and variability of data, unless stated otherwise.

## 3 RESULTS

### 3.1 Participant characteristics

There was a reduction in body mass following the FSITP in both groups during both experimental visits (Bimuno^®^ GOS: V1, −2.22% ± 0.48% and V2, −2.35% ± 0.67%; Placebo: V1, −2.44% ± 0.56% and V2, −2.33% ± 0.52%; *P* < 0.001), but this did not differ between day or group (*P* = 0.462). There were no differences in fluid intake (Bimuno^®^ GOS, 736 ± 245 mL; Placebo, 712 ± 242 mL; *P* = 0.591) or sweat rate, either between day or between group (Bimuno^®^ GOS: V1, 1.20 ± 0.34 L h^−1^ and V2, 1.28 ± 0.45 L h^−1^; Placebo: V1, 1.23 ± 0.39 L h^−1^ and V2, 1.20 ± 0.35 L h^−1^; *P* = 0.347). Supplement adherence was high, and there was no difference between groups (Bimuno^®^ GOS, 96% ± 5%; Placebo, 97% ± 2%; *P* = 0.301).

### 3.2 Absolute plasma I‐FABP

**FIGURE 3:**
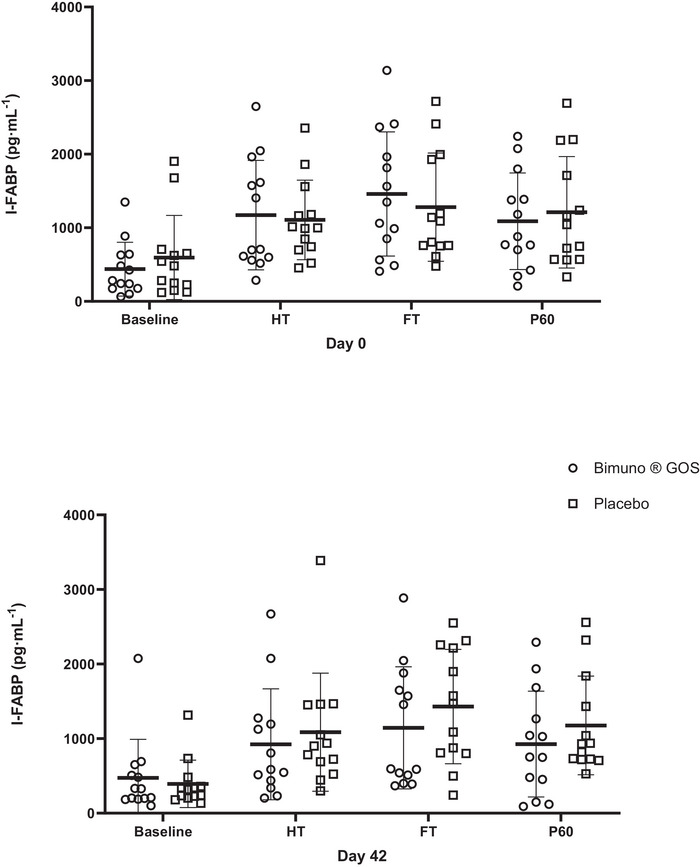
Plasma intestinal fatty acid binding protein (I‐FABP) (Bimuno^®^ GOS, *n* = 13, Placebo, *n* = 13) at baseline, half‐time (HT), full‐time (FT) and 60 min post (Post60) the football specific intermittent treadmill protocol at days 0 and 42. Values are the absolute value for each participant; midpoint line and whiskers represent the group mean ± SD.

For plasma I‐FABP concentration, there was no main effect of day [*F*(1, 24) = 1.943, *P* = 0.176, η^2^ = 0.075], but there was a difference over time [*F*(3, 72) = 23.222, *P* < 0.001, η^2^ = 0.492], with I‐FABP values being higher at half‐time, full‐time and 60 min post compared with baseline (*P* < 0.001). There was no day × time × group interaction effect [*F*(3, 72) = 2.529, *P* = 0.064, η^2^ = 0.095; Figure 3].

### 3.3 Peak ∆ plasma I‐FABP

**FIGURE 4:**
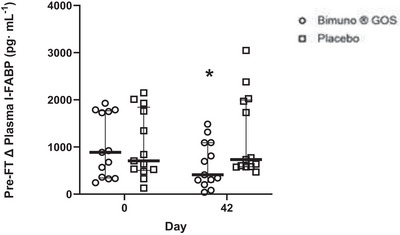
Pre‐full‐time (FT) ∆ plasma intestinal fatty acid binding protein (I‐FABP) at days 0 and 42 (Bimuno^®^ GOS, *n* = 13, Placebo, *n* = 13). A Kruskal–Wallis test revealed a significant between‐group difference in Pre‐FT ∆ plasma I‐FABP at day 42. Values are for each individual; bars represent the group median ± interquartile range. *Significant difference between groups during that visit (*P* < 0.05).

At D0 there was no between‐group difference in the pre‐full‐time ∆ in I‐FABP in response to the FSITP [χ^2^(1) = 0.024, *P* = 0.878] with a mean rank of 13.27 for Bimuno^®^ GOS and 13.73 for placebo (Figure 4). However, by D42 in the Bimuno^®^ GOS group the pre‐full‐time ∆ in I‐FABP following the FSITP had reduced by 38.6% compared with D0, which was a significantly greater change compared with that observed for the placebo group [χ^2^(1) = 3.995, *P* = 0.048], with a mean rank of 10.54 for the Bimuno^®^ GOS and 16.46 for placebo (Figure 4).

### 3.4 Absolute plasma LBP

**FIGURE 5:**
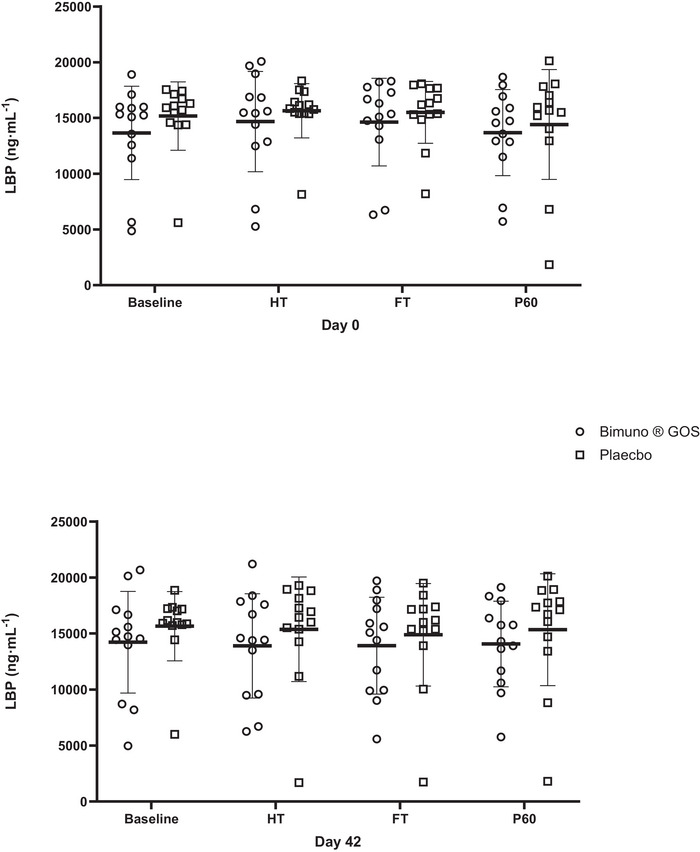
Plasma lipopolysaccharide binding protein (LBP) (Bimuno^®^ GOS, *n* = 13, Placebo, *n* = 13) at baseline, half‐time (HT), full‐time (FT) and 60 min post (Post60) the football specific intermittent treadmill protocol at days 0 and 42. Values are the absolute value for each participant; midpoint line and whiskers represent the group mean ± SD.

There was no main effect of day [*F*(1, 24) = 0.000, *P* = 0.996, η^2^ = 0.000], time [*F*(3, 72) = 0.284, *P* = 0.837, η^2^ = 0.492] or a day × time × group effect [*F*(3, 72) = 0.122, *P* = 0.947, η^2^ = 0.005] for plasma LBP (Figure 5).

### 3.5 Salivary IgA concentration

**FIGURE 6:**
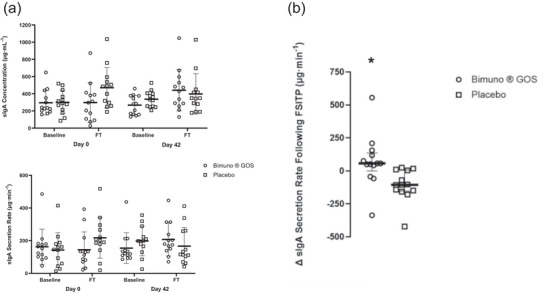
(a) Salivary IgA concentration (top) and salivary IgA (sIgA) secretion rate (bottom) (Bimuno^®^ GOS, *n* = 13, Placebo, *n* = 13) at baseline and full‐time (FT) during the football specific intermittent treadmill protocol on days 0 and 42. (b) The day 0 to day 42 ∆ sIgA secretion rate following football specific intermittent treadmill protocol. Student's unpaired *t*‐test revealed a significant between‐group difference (Bimuno^®^ GOS, *n* = 13, Placebo, *n* = 13). Values are peak ∆ for each participant; bars represent the group median ± interquartile range. *Significant difference between groups (*P* < 0.05).

The concentration of sIgA did not differ between day [*F*(1, 24) = 0.611, *P* = 0.442, η^2^ = 0.025], but there was a difference across time [*F*(3, 72) = 24, *P* = 0.009, η^2^ = 0.254] with sIgA concentration greater at full‐time compared with baseline (*P* = 0.009). There was a day × time × group interaction effect [*F*(1, 24) = 2.529, *P* = 0.006, η^2^ = 0.272; Figure 6a], but subsequent Student's unpaired *t*‐tests with Bonferroni correction revealed no between‐group differences at any time points (*P* < 0.05).

### 3.6 Salivary IgA secretion rate

The secretion rate of sIgA showed no main effect of day [*F*(1, 24) = 0.725, *P* = 0.403, η^2^ = 0.029] or time [*F*(1, 24) = 1.332, *P* = 0.260, η^2^ = 0.053], but there was a day × time × group interaction effect [*F*(1, 24) = 7.811, *P* = 0.010, η^2^ = 0.246; Figure 6a]. Student's unpaired *t*‐test with Bonferroni correction revealed no between‐group differences at any time points (*P* < 0.05). The post FSTIP change in sIgA secretion rate differed between groups at D42, with an increase observed in the Bimuno^®^ GOS group, whereas there was a reduction in the placebo group (Bimuno^®^ GOS, 74.00 ± 204.40 µg min^−1^; placebo, −106.19 ± 124.60 µg min^−1^; *P* = 0.016; Figure 6b).

### 3.7 URS

Throughout the 42 day intervention period there was no difference in the number of URS episodes between the Bimuno^®^ GOS and placebo groups (Bimuno^®^ GOS, 0.5 ± 0.5; placebo, 0.8 ± 0.4; *P* = 0.118). However, the average duration of a URS episode was shorter by 5.7 days in the Bimuno^®^ GOS group compared with the placebo group (Bimuno^®^ GOS, 3.2 ± 5.0 days; placebo, 8.9 ± 5.4 days; *P* = 0.007). The severity of reported URS episodes was lower in the Bimuno^®^ GOS compared with the placebo group (Bimuno^®^ GOS, 11.0 ± 16.1; placebo, 32.3 ± 21.2; *P* = 0.017). There was no between‐group difference in the number of training days affected by URS or the number of days of medication use (*P* > 0.05; Table 1).

**TABLE 1:** Overview of self‐reported upper respiratory symptom data during the 42 day intervention.

	B‐GOS (*n* = 13)		Placebo (*n* = 13)		
Parameter	Mean	SD	Mean	SD	*P‐*Value
Episode duration (days)	3.2	5.0	8.9	5.4	0.007
Incidence (episode per person)	0.5	0.5	0.8	0.4	0.118
Severity (symptom score per episode)	11.0	16.1	32.3	21.1	0.017
Training days affected by URS	0.4	1.1	1.3	2.3	0.178
Days of medication during URS	0.3	0.5	1.2	2.1	0.705

Abbreviations: B‐GOS, Bimuno‐galactooligosaccharide; URS, upper respiratory symptoms.

### 3.8 GIS during intervention period

There were no differences in the incidence of GIS across the 6 week intervention period (Bimuno^®^ GOS, 26.6 ± 24.5; Placebo, 30.5 ± 25.9; *P* = 0.428), but the Bimuno^®^ GOS group experienced fewer moderate GIS than placebo (Bimuno^®^ GOS, 0.31 ± 0.48; Placebo, 2.15 ± 3.18; *P* = 0.022). There was also a reduction in resting GIS severity from week 0 to week 6 in the Bimuno^®^ GOS group compared with the Placebo group (Bimuno^®^ GOS, −67.1% ± 36.1%; Placebo, −4.8 ± 111.1; *P* < 0.001).

### 3.9 GIS during exercise

**FIGURE 7:**
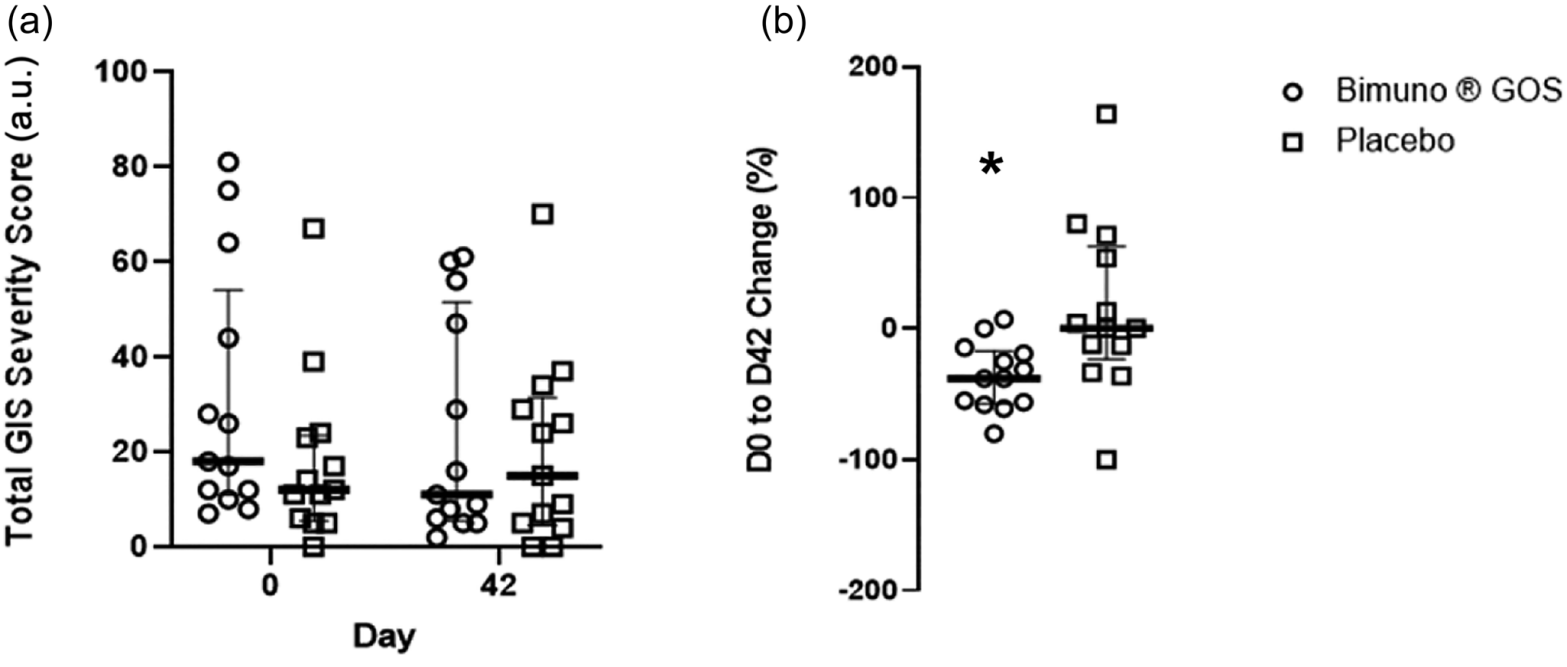
(a) Total gastrointestinal symptom (GIS) severity scores during football specific intermittent treadmill protocol at days 0 and 42 (Bimuno^®^ GOS, *n* = 13, Placebo, *n* = 13). (b) Percentage change in total GIS severity score during football specific intermittent treadmill protocol between days 0 and 42 (Bimuno^®^ GOS, *n* = 13, Placebo, *n* = 13). A Mann–Whitney *U*‐test revealed between‐group differences in the percentage change between days 0 and 42. Each data point represents a participant; bars represent the group median ± interquartile range. *Significant difference between groups (*P* < 0.05).

There was no between‐group difference in total GIS severity scores during exercise at D0 (*P* = 0.157; Figure 7a). There was a reduction in total GIS severity score at D42 compared with D0 during the FSITP for the Bimuno^®^ GOS group but not Placebo (Bimuno^®^ GOS, −38.9% ± 24.2%; Placebo, 14.8% ± 65.4%; *P* = 0.009; Figure 6b). For GIS subcategories, there was a reduction in the severity score for ‘other GIS’ from D0 to D42 in the Bimuno^®^ GOS group but not the placebo group (Bimuno^®^ GOS, −34.6% ± 54.8%; Placebo, 32.5% ± 96.5%; *P* = 0.006). There was no between‐group difference for the D0 to D42 change in upper GIS severity scores (Bimuno^®^ GOS, −46.7% ± 33.4%; Placebo, −16.8 ± 84.1; *P* = 0.337) or lower GIS severity scores (Bimuno^®^ GOS, −25.9% ± 64.8%; Placebo, 36.7 ± 85.9; *P* = 0.175). There was also no difference between groups for the change in symptom incidence between D0 and D42 (Bimuno^®^ GOS, −0.5 ± 5.4; Placebo, −1.3 ± 3.9; *P* = 0.977).

### 3.10 Physiological and subjective responses to the FSITP

Mixed‐model ANOVAs revealed increases in *T*_core_, HR, RPE, thermal sensation and subjective fatigue over the course of the FSITP (*P* < 0.001; Table 2). However, for these variables there were no differences between groups or days (*P* > 0.05). A mixed‐model ANOVA revealed that lactate increased over FSITP (*P* < 0.001, η^2^_p_ = 0.449) and that there was a difference between group (*P* = 0.007, η^2^_p_ = 0.285), with lower lactate concentrations in Bimuno^®^ GOS than Placebo at full‐time on D42 (Bimuno^®^ GOS, 1.69 ± 0.62 mmo L^−1^; Placebo, 2.45 ± 0.62; *P* = 0.007; Table 2).

**TABLE 2:** Overview of physiological responses during football specific intermittent treadmill protocol at D0 and D42 visits in B‐GOS and Placebo groups.

	B‐GOS (*n* = 13)		Placebo (*n* = 13)	
Outcome	D0	D42	D0	D42
Heart rate (beats min^−1^)	152 (13)	151 (17)	151 (12)	155 (12)
∆Peak core temperature (°C)	2.1 (0.5)	2.0 (0.5)	2.0 (0.5)	2.1 (0.4)
RPE	13 (2)	13 (2)	13 (2)	13 (1)
Lactate (mmol L^−1^)	1.9 (0.6)	1.7 (0.6)	2.4 (0.6)	2.5 (0.6)

*Note*: Data are presented as the mean (SD). ∆Peak core temperature is the peak change from baseline value. Abbreviations: B‐GOS, Bimuno‐galactooligosaccharide; D, day; RPE, rating of perceived exertion; URS, upper respiratory symptoms.

## 4 DISCUSSION

The aim of the present study was to evaluate the effects of a 42 day prebiotic (Bimuno^®^ GOS) intervention on GIS, circulatory markers of intestinal epithelial injury and permeability, and immunity during a simulated football activity in the heat, whilst also assessing URS and GIS over the intervention period. The present study is thought to be the first to demonstrate that 42 days of the daily prebiotic Bimuno^®^ GOS (2.75 g day^−1^ active GOS) can attenuate the increase in circulating I‐FABP after football‐based exercise in the heat, suggestive of improved gut barrier integrity, whilst lowering exercise‐induced GIS severity. Furthermore, 42 days of Bimuno^®^ GOS reduced resting GIS severity. Finally, those supplemented with Bimuno^®^ GOS experienced shorter and less severe URS episodes during the intervention period, and there was a greater maintenance of sIgA secretion rate post‐exercise at D42. Collectively, these findings demonstrate that supplementation with Bimuno^®^ GOS might be beneficial for team‐sport‐based athletes during match‐play and training periods owing to alleviation of GIS and damage, in addition to providing immune support, as evidenced by increased sIgA secretion rates and attenuation of URS.

Acute illness is the second most common reason for an athlete to require medical attention (Palmer‐Green et al., 2013). In team sports such as rugby and football, both GI and upper respiratory disturbances contribute significantly to absences and impaired performance (Cunniffe et al., 2009; Parry & Dust 2006; Wilson et al., 2023). Team‐based athletes face numerous challenges, including high workload, frequent competitive fixtures, psychological stress, environmental stress, poor sleep and travel, all of which might temporarily depress immune function and increase the risk of acute GIS and URS (Walsh, 2018). Evidence suggests that exercising in hotter ambient temperatures increases the incidence of GIS (Costa et al., 2017). This is a concern for team‐sport athletes because it is becoming increasingly common for major tournaments and pre‐season periods to be played in such climates. Although the majority of GIS were mild, we still saw a reduction during exercise after 42 days of Bimuno^®^ GOS and a lower incidence of moderate GIS during the supplementation period. This demonstrates that Bimuno^®^GOS might be a suitable intervention for athletes looking to alleviate GI discomfort. This is in accordance with previous work demonstrating reductions in the severity and incidence of GIS in elite rugby union players following a 24 week supplementation with Bimuno^®^ GOS (Parker et al., 2023). The novelty of the present study is that GIS improvements occurred both during a specific period of exercise and after a shorter intervention period.

In addition to reduced GIS, we observed a 39% reduction in pre‐full‐time Δ plasma I‐FABP during the FSITP after the 42 day Bimuno^®^ GOS intervention, indicating greater intestinal barrier resistance. Intestinal epithelial barrier disruption occurs during exercise as blood flow is redistributed away from the splanchnic region to aid skeletal muscle perfusion (Reher et al., 2001), resulting in ischaemic stress. I‐FABP is a cytosolic enterocyte protein that is abundant in the intestinal mucosa and is used as an indirect marker of intestinal damage. Elevated levels of I‐FABP have been reported consistently following steady state and, more recently, competitive team‐sport based exercise (Chantler et al., 2022; Clayton et al., 2024). The levels seen in the present study are similar to those reported by Chantler et al. (2022) and Clayton et al. (2024) but also those at rest in patients with Crohn's disease (1339 pg mL^−1^) and ulcerative colitis (1309 pg mL^−1^) (Logan et al., 2022). It is unclear whether the elevations observed are clinically significant and likely to invoke clinical symptoms because I‐FABP began to decrease towards baseline values by P60 and did not persist like those seen in the aforementioned conditions. However, the 39% reduction following Bimuno^®^ GOS intervention is still an interesting finding because it might help to alleviate other complications associated with exercise‐induced intestinal barrier damage for athletes, such as GIS and orocaecal transit time (Gaskell et al., 2023) and nutrient malabsorption (van Wijck et al., 2013), in addition to providing the scope for future studies investigating the impact of Bimuno^®^ GOS on resting and exercising concentrations in individuals with gastrointestinal illnesses.

Our findings confirm those of Rauch et al. (2025), who observed a modest attenuation of I‐FABP in response to 180 min of running at 60% of ${\dot{V}}_{O_{2}\max}$ in 30°C heat following an 8 week prebiotic intervention. Interestingly, their intervention was 16 g day^−1^ of a prebiotic mixture including guar gum, inulin, green banana, sweet potato starch and β‐GOS. Thus, our results confirm that it is possibly the GOS element of the supplement that influences the intestinal barrier, and such benefits are still present when a smaller dose is administered over a shorter period. In murine studies, GOS reduced lipopolysaccharide‐induced intestinal damage and inflammation, enhancing epithelial integrity by increasing villus height, villus‐to‐crypt ratio and the expression of tight junction proteins (ZO‐1, occludin and claudin‐1) (Wang et al., 2021). GOS might also promote intestinal development through effects on cell maturation, nutrient absorption (Cheng et al., 2018; Hu et al., 2012) and goblet cell function. Specifically, GOS upregulates MUC2 and trefoil factor genes involved in mucosal barrier maintenance (Bhatia et al., 2015).

Prebiotic Bimuno^®^ GOS has previously been shown to be highly bifidogenic (Depient et al., 2008), increasing the growth of beneficial bifidobacteria and supporting the maintenance of gut homeostasis whereby the proliferation of beneficial bacteria might increase competition and inhibit the colonization of pathogenic bacteria. GOS supplementation also increases other beneficial gut bacteria, including *Lactobacillus johnsonii*, *Lactobacillus murinus*, *Lactobacillus reuteri* and *Akkermansia muciniphila*, the last of which enhances mucin production and supports epithelial integrity (Wang et al., 2022; Zou et al., 2020). These microbiome shifts promote short‐chain fatty acid production, specifically increasing acetate, propionate and butyrate, which support gut barrier function, immune modulation and pathogen inhibition (Del Fabbro et al., 2020). GOS has been shown to preserve short‐chain fatty acid levels during inflammatory challenges, contributing to improved barrier resistance (Wang et al., 2021). Short‐chain fatty acids also activate G‐protein‐coupled receptor 43 in endocrine L cells, stimulating intestinal epithelial cell production and maintaining mucosal integrity (Connor et al., 2016; Dube & Brubaker, 2007; Fernandez et al., 2016; Oozeer et al., 2013). Thus, Bimuno^®^ GOS might enhance intestinal resilience to exercise‐induced stress via microbiome‐mediated and morphological mechanisms, potentially reducing local inflammation and GIS.

Alongside reductions in GIS, we observed a 5.7 day reduction in the duration of URS episodes and a lower severity in the Bimuno^®^ GOS group compared with placebo. Similar findings were found in elite rugby union players when using the same supplement over the course of 24 weeks (Parker et al., 2023). Interestingly, we have shown that improvements are evident as early as 6 weeks, which might be important when athletes are preparing for significant events. The use of non‐digestible oligosaccharides for repressing URS has received some attention. In double‐blind controlled trials, the combination of GOS and Fructooligosaccharides and GOS alone were able to reduce the incidence and recurrence of upper respiratory infections in infants during the first 6–12 months of life (Arslanoglu et al., 2007; Maldonado et al., 2012). In a group of stressed students, 2.5 and 5 g day^−1^ doses of GOS reduced the percentage of days URS was present (Hughes et al., 2011). Similar to the effects of GOS on barrier integrity, the proposed mechanisms for reducing URS are changes in microbiome composition and an increase in short‐chain fatty acid production. Butyrate has been shown to increase the production of antimicrobial peptides in the lung epithelial cell line VA10 (Steinmann et al., 2009). Acetate is also reported to have protective effects against influenza and pulmonary infections in mice (Sencio et al., 2020). Increasing evidence suggests that non‐digestible oligosaccharides have the potential to be absorbed into the systemic circulation after oral administration, enabling them to reach the lungs and have direct effects on pathogens within airway epithelial cells (Vazquez et al., 2017; Ruhaak et al., 2014). Such interactions might also impact inflammation, nuclear factor‐κB activation and cytokine/chemokine production (Mussatto & Mancilha, 2007). For example, GOS has been shown to inhibit *Mannheimia haemolytica*‐induced cytokine/chemokine production, TLR‐4 expression and the associated mitogen‐activated protein kinase/nuclear factor‐κB pathway, in addition to reducing the lipopolysaccharide (TLR‐4 ligand)‐induced cytokine/chemokine release in calf primary bronchial epithelial cells, possibly reducing the onset of URS (Cai et al., 2022).

This is the first study to explore the effects of a lower dose of galactooligosaccharide prebiotic on exercise‐induced GI damage, symptoms and immunity. One benefit of replicating a football match in a laboratory environment is that distance, intensity, ambient temperature and water intake were matched at D0 and D42. Conversely, it meant that we were unable to simulate movements specific to competitive football matches, including changes in direction, jumping and contact. It is unclear how much this might influence gut barrier damage and circulating I‐FABP, but the reported values in the present study support increases in I‐FABP following a professional football match (Clayton et al., 2024). There were several limitations with the study design. Although we did ensure that participants did not complete the experiment between October and May to avoid the warmer months and limit heat acclimatization, we could not guarantee temperature fluctuations over the 6 week periods that might have influenced responses, and not all participants completed the study during the same 6 week period. Furthermore, although participants were instructed to avoid any dietary supplements and changes in diet and training during the 6 weeks, this was not logged; participants were only instructed to follow the same diet in the 24 h preceding each visit. Given that GIS and URS were self‐reported throughout the study, it is possible that some symptoms were non‐infection related. Although the methods of assessment have been validated to infection, this cannot be guaranteed. Another limitation was the lack of inflammatory markers, and the assessment of the microbiome and short‐chain fatty acid production. It is possible that Bimuno^®^ GOS improved intestinal barrier integrity and reduced GIS and URS via pathways that involved these elements. Furthermore, given that we have shown that Bimuno^®^ GOS can reduce acute respiratory and GI symptoms in both field and laboratory‐based studies, future research should be conducted to establish the underpinning mechanisms. Finally, the lack of a power calculation might have blunted the statistical power of the study. This omission was attributable to no prior literature on prebiotics and exercise‐induced GI barrier integrity. Future research should use the findings in the present study to perform such calculations.

## 5 CONCLUSION

In conclusion, a 42 day supplementation with prebiotic Bimuno^®^ GOS reduced exercise‐induced GI damage and GIS during a simulated football match in the heat. This was accompanied by a greater maintenance of sIgA immediately postexercise. In accordance with previous literature, Bimuno^®^ GOS also reduced URS severity and duration in addition to GIS during the supplementation period. These findings suggest that prebiotic Bimuno^®^ GOS might have the potential to enhance GI barrier integrity during exercise, modulate immune function and reduce the burden of acute respiratory illness and GI symptoms, which might improve athlete availability. Future research should explore the possible underpinning mechanisms of Bimuno^®^ GOS on the alleviation of illness and exercise‐induced GI damage.

## AUTHOR CONTRIBUTIONS

Connor J. Parker, Graham R. Sharpe, Michael A. Johnson, Kirsty A. Hunter and Neil C. Williams contributed to the conceptualization and design of the study. Connor J. Parker, Samantha J. Abbott, Luke R. Butterfield and Neil C. Williams were responsible for data collection. Connor J. Parker and Neil C. Williams were responsible for data analysis. All authors reviewed the article and provided critical feedback. All authors approved the final version of the manuscript and agree to be accountable for all aspects of the work in ensuring that questions related to the accuracy or integrity of any part of the work are appropriately investigated and resolved. All persons designated as authors qualify for authorship, and all those who qualify for authorship are listed.

## CONFLICT OF INTEREST

The authors declare no conflicts of interest.

## FUNDING INFORMATION

None.

## Data Availability

The data presented in this study are available on request from the corresponding author. The data are not publicly available for reasons of privacy.
